# Supplementary material for: Coarse-grained versus fully atomistic machine learning for zeolitic imidazolate frameworks
Source: Chem Commun (Camb). 2023 Aug 22;59(76):11405–8. doi: 10.1039/d3cc02265j (PMC10513772; doi:10.1039/d3cc02265j)
Supplement: CC-059-D3CC02265J-s001 [file CC-059-D3CC02265J-s001.pdf]

Electronic Supplementary Material (ESI) for ChemComm.
This journal is © The Royal Society of Chemistry 2023

Electronic Supplementary Information (ESI) for

Coarse-grained *versus* fully atomistic machine learning for zeolitic imidazolate frameworks

Zoé Faure Beaulieu, Thomas C. Nicholas, John L. A. Gardner, Andrew L. Goodwin,* and Volker L. Deringer*

* andrew.goodwin@chem.ox.ac.uk; volker.deringer@chem.ox.ac.uk

This ESI document contains:

Supplementary discussion (I): Dataset **S2**

Supplementary discussion (II): Hyperparameters and numerical errors **S5**

Supplementary discussion (I): Dataset

Data source. The data source for our study is an empirical force-field model (MOF-FF parameterised as described in Ref. 14). To justify the use of this particular model in our work, we show in Fig. S1 that the majority of our dataset lies well within the range tested by the MOF-FF model used. We acknowledge that a few structures fall outside this range; however, these are rare occurrences and their impact on the results is expected to be small.

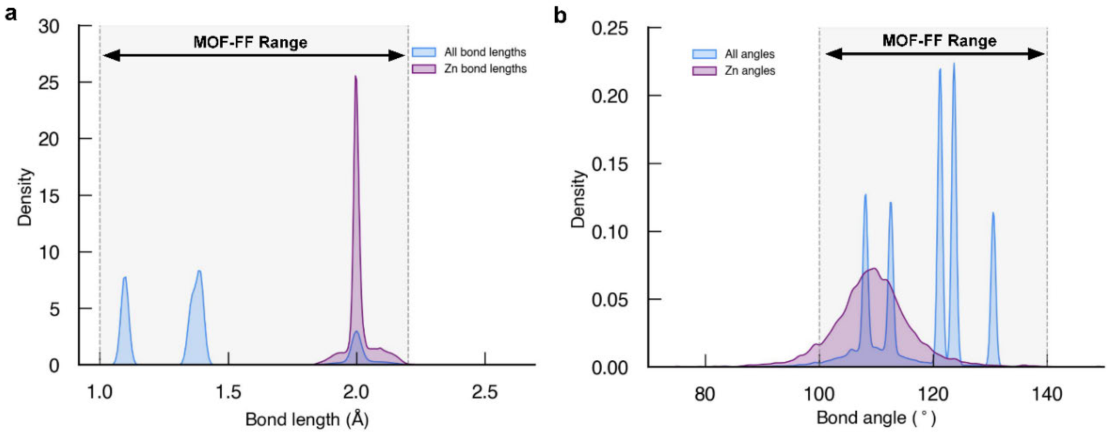

Fig. S1. Distribution of structural properties in our database compared to the range tested for the MOF-FF model (light grey shading) in Ref. 14. (**a**) Distribution of bond lengths. (**b**) Distribution of bond angles. The blue plots show the distribution of bond lengths / angles for all atoms, whereas the purple plots contain only bond lengths involving Zn^{2+} / angles with Zn^{2+} as the central atom.

Structure generation. The structures were generated by decorating ("back-mapping") a set of AB_2 networks taken from prior work; the details of the structure generation procedure are described at https://github.com/tcnicholas/hZIF-data. One central aspect of the dataset generation is the distortion ("rattling") of structures, taking them slightly away from the (MOF-FF-optimised) local-minimum configurations. This is in analogy to common practice in the fitting of ML potential models.

The dataset contains structures with different degrees of those perturbations, labelled "`small`", "`medium`", and "`large`". We use two different datasets to fit the GPR models:

- A "main" dataset, containing only the "`medium`"-distorted configurations such that all structures are generated in a consistent way. We show fitting results for this dataset in the main text, and in Fig. S2a we additionally test models fitted to that dataset on structures with "`small`" and "`large`" distortions, respectively.
- An "extended" dataset, containing all of the "`small`"-, "`medium`"-, and "`large`"-distorted configurations. We show in Figs. S2b and S3 that qualitatively similar conclusions can be drawn from fitting to this extended dataset.

We note the appearance of some clusters of points away from the diagonal (Fig. S2); we speculate that those might be due to structures with too little "rattling" that are therefore locally too similar to one another. Further work is required, however, for a full assessment.

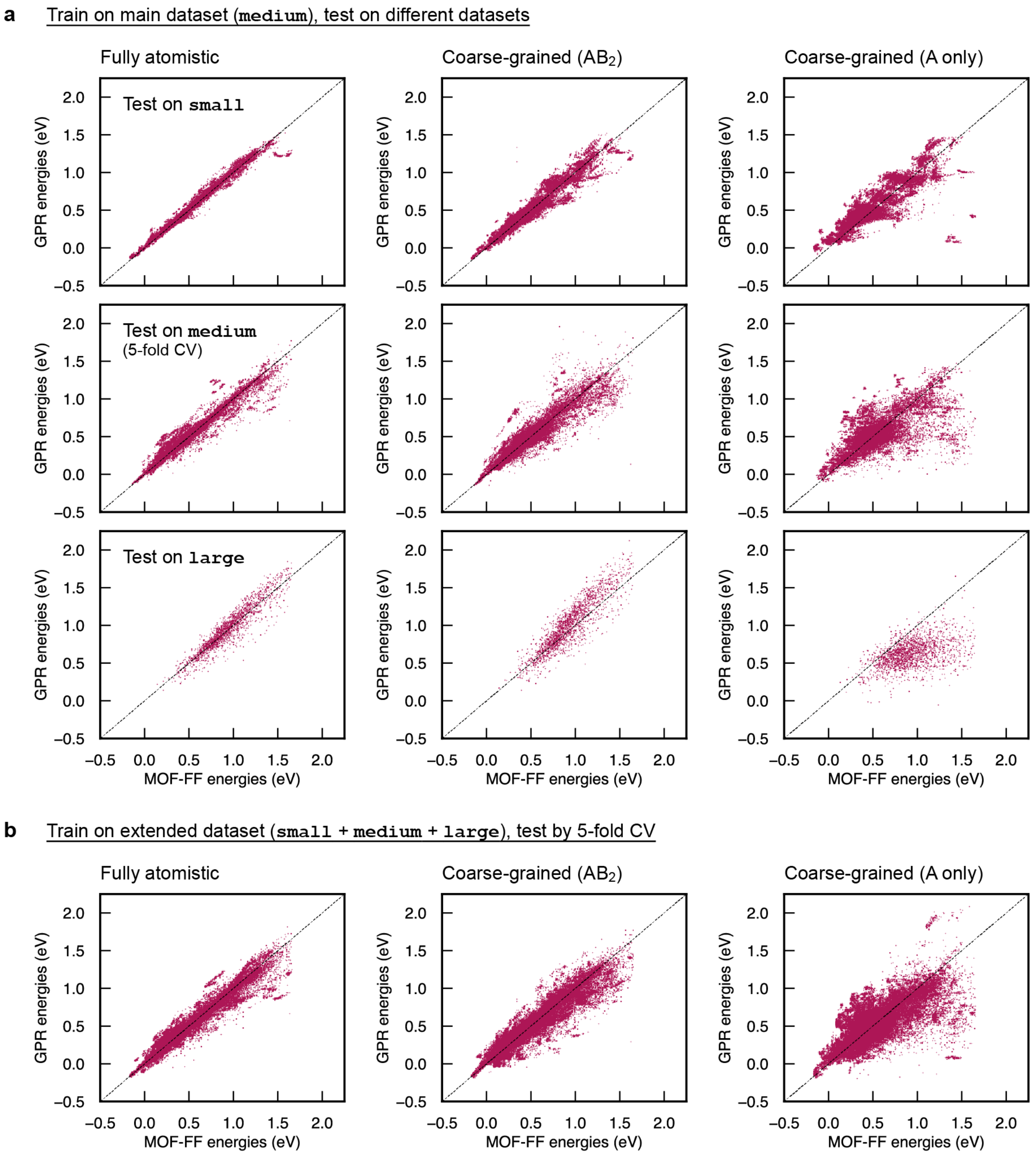

Fig. S2 *(as a supplement to Fig. 2b in the main text)*. Scatter plots of local-environment energies as defined in the main text and the associated GPR ML predictions. From left to right, we characterise GPR models based on: a fully atomistic description; a cg description where the linker molecules are described by single "B" beads; and one where only A-site species are represented. (**a**) Tests for the GPR model described in the main text, fitted using 32,000 data points from the main ("`medium`") dataset. We show tests for this model, from top to bottom: on structures with smaller distortions than in the training; on the same dataset (using 5-fold cross-validation), as shown in Fig. 2b; and on structures with larger distortions than in the training. (**b**) As before, but now for training and 5-fold cross-validation for the extended dataset containing all relevant configurations. Whilst the absolute errors and the details in the appearance of the scatter plots differ from case to case, the overall interpretation is similar: cg models based on an AB_2 representation (*middle*) perform reasonably well compared to the fully atomistic ones; cg models based on an A-site only representation (*right*) perform less well.

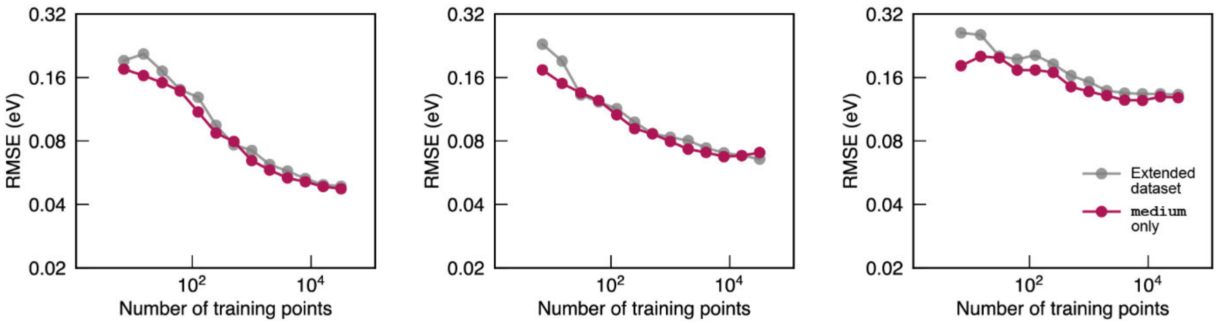

Fig. S3 *(as a supplement to Fig. 2c in the main text)*. Comparison of learning curves for the extended dataset (grey) and the main dataset (`medium` only, as shown in the main text; magenta). The learning curves are qualitatively similar and lead to essentially unchanged conclusions irrespective of which dataset is used for fitting, consistent with our interpretation of Fig. S2. All errors were obtained by 5-fold cross-validation.

Supplementary discussion (II): Hyperparameters and numerical errors

Our Communication shows cg-GPR models using the Smooth Overlap of Atomic Positions (SOAP) approach to construct the descriptors; see Ref. 20 in the main text.

Angular basis functions. The SOAP parameters used in Fig. 2c are n_{max} = 16 and l_{max} = 8, where n_{max} and l_{max} control the number of radial and angular basis functions used, respectively. Here, we perform an ablation test by gradually reducing l_{max} to 0, which is equivalent to removing angular information from the local-environment featurisation.

Figure S4 shows the results of this test for all three classes of structures (atomistic, AB_2 cg and A-only cg), evaluated for the extended dataset. As expected, comparison with l_{max}=8 (pink) clearly shows that the model performs significantly worse when the descriptors neglect angular features. This is particularly acute in the case of the atomistic model. As the degree of coarse-graining increases, the difference between the models decreases: once we have reached an A-site only model, adding additional angular information provides little benefit given that there are much fewer degrees of freedom to consider in the first place.

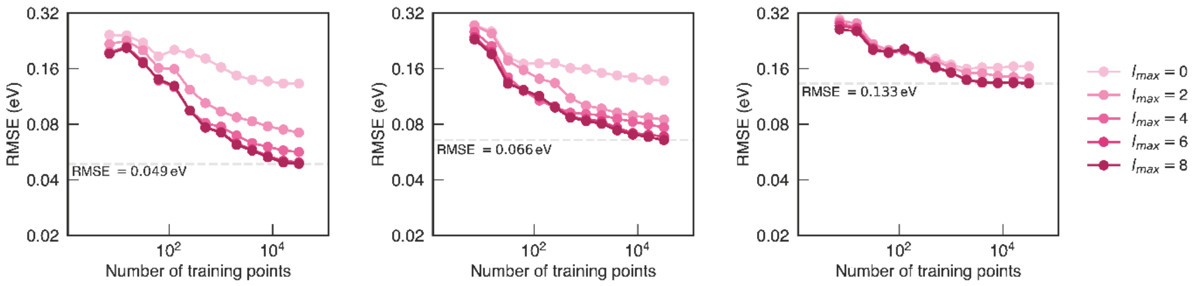

Fig. S4. Learning curves showing the model root mean square error (RMSE) depending on the number of training datapoints for lowering l_{max}. The results from l_{max}=8 (also shown in grey in Fig. S3) were added for direct comparison. The RMSE for the largest number of training points is indicated by a dashed grey line in each panel. From left to right, we characterise GPR models based on a fully atomistic description; a cg description where the linker molecules are described by single “B” beads; and a more aggressively coarse-grained model where only the A-site species are represented.

Cut-off and smoothness. In Fig. 3 of the main text, we scan the hyperparameter space defined by the radial cut-off and the smoothness used in constructing the atomic neighbour density. We re-computed this scan using the extended dataset and compare both plots in Fig. S5. The optimised hyperparameters for all models are listed in Table S1.

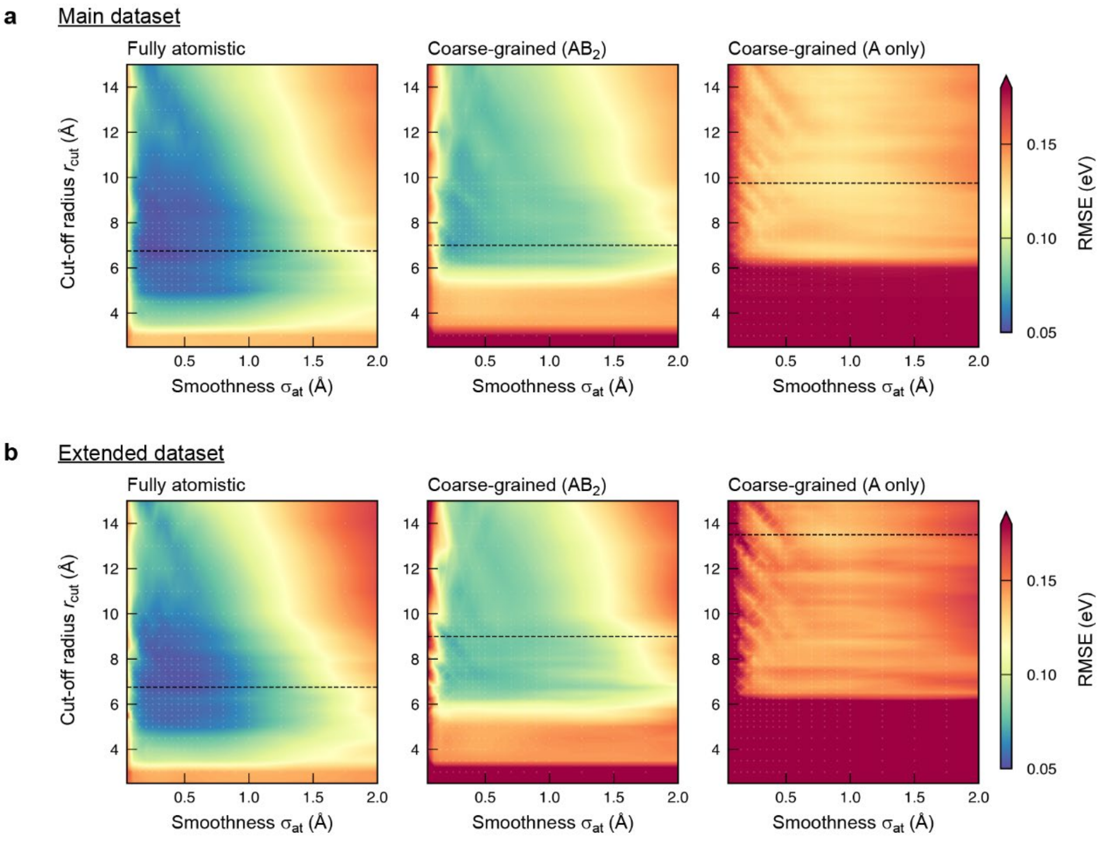

Fig. S5 *(as a supplement to Fig. 3 in the main text)*. As Fig. 3, but now comparing results for the main and extended datasets (see above), and indicating the respective values of the optimised cut-off radii by dashed horizontal lines.

Table S1. Optimised SOAP hyperparameters obtained via grid searches using *N* = 10,000 training datapoints (*cf.* Fig. S5 above). Note that the above surfaces are very shallow, especially in terms of the σ_{at} dependence, and therefore those “optimised” values are only given for completeness.

	**Main dataset**		**Extended dataset**	
	r_{cut} (Å)	σ_{at} (Å)	r_{cut} (Å)	σ_{at} (Å)
Fully atomistic	6.75	0.30	6.75	0.50
cg (AB_2)	7.00	0.25	9.00	0.20
cg (A only)	9.75	0.90	13.50	0.80

Hyperparameter transferability. Supporting the analysis discussed in the main text, we cross-checked how using the optimised hyperparameters for one representation affects the prediction error of another (Table S2). The effect of optimising the hyperparameters is notable for fully atomistic models, less strongly so for the cg ones.

Table S2. RMSEs for different GPR models for local-environment energies in ZIFs. The columns show results for the three different representations. The rows correspond to hyperparameters, $\mathcal{H}$, optimised for the respective representation (*cf.* Table S1).

	**Main dataset**, RMSE for $\varepsilon_{\text{local}}^{(i)}$ (eV)		
	Fully atomistic	cg (AB_2)	cg (A only)
$\mathcal{H}$ for atomistic	**0.047**	0.074	0.138
$\mathcal{H}$ for cg (AB_2)	0.047	**0.071**	0.135
$\mathcal{H}$ for cg (A)	0.063	0.078	**0.128**
	**Extended dataset**, RMSE for $\varepsilon_{\text{local}}^{(i)}$ (eV)		
	Fully atomistic	cg (AB_2)	cg (A only)
$\mathcal{H}$ for atomistic	**0.049**	0.075	0.144
$\mathcal{H}$ for cg (AB_2)	0.054	**0.066**	0.157
$\mathcal{H}$ for cg (A)	0.082	0.083	**0.133**

Zn^{2+} *versus* local-environment energies. We also tested the errors for GPR models that regress only the MOF-FF energies of the Zn^{2+} environments themselves (Table S3). The magnitude of the errors is lower, but the trends observed are similar to those for the local-environment energies (as discussed in the main text). We also performed the same analysis on the extended dataset (see above), again leading to similar conclusions as for the main one.

Table S3. RMSEs for GPR models of the local-environment energies (the "Main dataset" results are the same as the values shown in Fig. 2c of the main text), and for GPR models of the Zn^{2+} energies only – that is, not considering the atomic energies of the atoms in the linkers.

	**Main dataset**, cross-validation RMSE (eV)		
	Fully atomistic	cg (AB_2)	cg (A only)
Local environment, $\varepsilon_{\text{local}}^{(i)}$	0.047	0.071	0.128
Zn^{2+} energies only	0.011	0.016	0.048
	**Extended dataset**, cross-validation RMSE (eV)		
	Fully atomistic	cg (AB_2)	cg (A only)
Local environment, $\varepsilon_{\text{local}}^{(i)}$	0.049	0.066	0.133
Zn^{2+} energies only	0.013	0.018	0.053
